# Evaluating the Psychometric Properties of the Maslach Burnout Inventory-Human Services Survey (MBI-HSS) among Italian Nurses: How Many Factors Must a Researcher Consider?

**DOI:** 10.1371/journal.pone.0114987

**Published:** 2014-12-12

**Authors:** Barbara Loera, Daniela Converso, Sara Viotti

**Affiliations:** Department of Psychology, University of Turin, Turin, Italy; University of Perugia, Italy

## Abstract

**Background:**

The Maslach Burnout Inventory (MBI) is the mainstream measure for burnout. However, its psychometric properties have been questioned, and alternative measurement models of the inventory have been suggested.

**Aims:**

Different models for the number of items and factors of the MBI-HSS, the version of the Inventory for the Human Service sector, were tested in order to identify the most appropriate model for measuring burnout in Italy.

**Methods:**

The study dataset consisted of a sample of 925 nurses. Ten alternative models of burnout were compared using confirmatory factor analysis. The psychometric properties of items and reliability of the MBI-HSS subscales were evaluated.

**Results:**

Item malfunctioning may confound the MBI-HSS factor structure. The analysis confirmed the factorial structure of the MBI-HSS with a three-dimensional, 20-item assessment.

**Conclusions:**

The factorial structure underlying the MBI-HSS follows Maslach’s definition when items are reduced from the original 22 to a 20-item set. Alternative models, either with fewer items or with an increased number of latent dimensions in the burnout structure, do not yield better results to justify redefining the item set or theoretically revising the syndrome construct.

## Introduction

Occupational burnout is a psychological response to chronic work-related stress of an interpersonal and emotional nature that appears in professionals working directly with clients, patients, or other recipients. Maslach defined burnout in the 1970s as “a syndrome of emotional exhaustion, depersonalization, and reduced personal accomplishment that can occur among individuals who do ‘people work’ of some kind” ([1], p. 3). This conceptualization led to the identification of the three main dimensions of burnout that are assessed in the Maslach Burnout Inventory-MBI [2], the worldwide leading instrument for the assessment of burnout, by means of three sub-scales: *emotional exhaustion* (EE), *depersonalization* (DP), and *personal accomplishment* (PA).

Various versions of the MBI exist. The first [3], intended for workers employed in health and social services, was later renamed MBI-Human Service Survey (MBI-HSS) to differentiate it from the one developed for educators, the MBI-Educators’ Survey (MBI-ED) [1]. In the 1990s, research on burnout was extended to professionals other than those employed in human services: Schaufeli *et al.* [4] developed a third questionnaire, the MBI-General Survey (MBI-GS), to be used for general professionals.

Despite its popularity, the validity of the MBI – in all its versions – has been the subject of considerable debate [5], [6], [7] and many scholars tested alternative models of the inventory, increasing or decreasing the number of the factors or reducing the original 22-item set. The studies conducted between 2000 and 2014 on the factorial structure and on the psychometric properties of the MBI-HSS are systematically reviewed and described in Table 1 in terms of samples, data analysis, and results.

**Table 1:** Systematic review of the main studies on MBI-HSS (2000–2014).

Study number/Year	Author(s)	Analysis	Final model	Final model fit	Data
(1) 2000	Kalliath *et al.*	CFA (ML) and measurement invariance across 3 groups	**7 items** (deleted: 2, 4, 5, 6, 7, 8, 9, 12, 15, 16, 17, 18, 19, 21, 22). **2 factors: EE**: 1, 3, 13, 14, 20 (α = .83); **DP:** 10, 11 (α = .89). Correlations: r _EE*DP_ = .50	**χ^2^** = 13.26; **df** = 13; **χ^2^/df** = 1.02; **CFI** = 1.00; **RMSEA** = .01	197 nurses, 113 laboratory technicians, and 135 managers. Midwest, US.
(2) 2001	Densten	EFA+ CFA (estimation method non indicated) validation on two subsamples obtained splitting the original sample by a random procedure	**19 items** (deleted: 12, 13, 14). **5 factors:** **EE_SS**: 1, 2, 3, 8 (α = .84); **EE_PS**: 6, 16, 20 (α = .90); **DP**: 5, 10, 11, 15, 22 (α = .73); **PA_S**: 4, 9,18, 19 (α = .70); **PA_O**: 7, 17, 21 (α = .58). Correlations: r_EE_SS*EE_PS_ = .69; r_EE_SS*DP_ = .55; r_EE_PS*DP_ = .48; r_EE_SS*PA_S_ = −.33 r_EE_SS*PA_O_ = −.27; r_EE_PS*PA_S_ = −.30 r_EE_PS*PA_O_ = −.21; r_DP*PA_S_ = −.27; r_DP*PA_O_ = −.17; r_PA_S*PA_O_ = .40	*n_1_*: **χ^2^** = 197.87; **df** = 109; **χ^2^/df** = 1.82; **CFI** = .94. *n_2_*: **χ^2^** = 219.96; df = 109; **χ^2^/df** = 2.02; **CFI** = .92	480 law enforcement management workers. Australia.
(3) 2001	Schaufeli *et al.*	CFA (ML) and measurement invariance across 2 groups	**20 items** (deleted: 12, 16). **3 factors:** **EE**: 1, 2, 3, 6, 8, 13, 14, 20 (α = .89); **DP**: 5, 10, 11, 15, 22 (α = .67); **PA**: 4, 7, 9, 17, 18, 19, 21 (α = .75). Correlations: r_EE*DP_ = ni, r_EE*PA_ = ni, r_DP*PA_ = ni. N.B.12 covariations between error terms within the sets of negatively (EE, DP) and positively (PA) phrased items]	**χ^2^** = 443.09; **df** = 310; **χ^2^/df** = 1.43; **CFI** = .89; **RMSEA** = .06	139 workers employed in different sectors outpatients from a psychotherapeutic treatment (n_not-burned_ = 68; n_burned_ = 71). Nederland.
(4) 2002	Beckstead	CFA (ML)	**22 items** (no delete items), **3 factors: EE**:1, 2, 3, 6, 8, 12, 13, 14, 16, 20 (α = .88). **DP**: 5, 10, 11, 15, 16, 22 (α = .80); **PA**: 4, 7, 9, 12, 17, 18, 19, 21 (α = .75). Correlations: r_EE*DP_ = .65; r_EE*PA_ = −.16; r_DP*PA_ = −.25. N.B. 23 covariations between error terms and cross-loadings of items 12 (on EE and PA) and 16 (on EE and DP).	**χ^2^** = 192.78; **df** = 180; **χ^2^/df** = 1.07; **CFI** = .99; **RMSEA** = .02	151 registered nurses. West-central Florida, US.
(5) 2004	Richardsen & Martinussen	CFA (ML) and measurement invariance across 7 groups	**20 items** (deleted: 12 and 16), **3 factors**: **EE:** 1, 2, 3, 6, 8, 13, 14, 20 (α = .90). **DP:** 5, 10, 11, 15, 22 (α = .66). **PA:** 4, 7, 9, 17, 18, 19, 21 (α = .77). Correlations: r_EE*DP_ = .46; r_EE*PA_ = −.05; r_DP*PA_ = −.20	**χ^2^** = 2722.37; **df** = 1271; **χ^2^/df** = 2.14; **CFI** = .88; **RMSEA** = .03	1590 workers of seven different profession (healthcare, social and educational sector). Norway.
(6) 2004	Hallberg & Sverke	CFA (ML) + cross-validation on two subsamples	**20 items** (deleted: 12 and 16), **3 factors: EE:** 1, 2, 3, 6, 8, 13, 14, 20 (α_n1_ = .87; α_n2_ = .84), **DP:** 5, 10, 11, 15, 22 (α_n2_ = .78; α_n2_ = .75), **PA:**4, 7, 9, 17, 18, 19, 21(α_n1_ = .75; α_n2_ = .75). Correlations: r_n1EE*DP_ = .60, r_n2EE*DP_ = .56; r_n1EE*PA_ = .17, r_n2EE*PA_ = .08; r_n1DP*PA_ = .36 r_n2DP*PA_ = .33	**χ^2^** = 894.54; **df** = 377; **χ^2^/df** = 2.4; **CFI** = .96; **RMSEA** = .06	Two healthcare samples (n_1_ = 544; n_2_ = 462). Sweden.
(7) 2005	Gil-Monte	CFA (WLS) validation on two subsamples obtained splitting the original sample by a random procedure	**20 items** (deleted: 12 and 16), **4 factors:** **EE:** 1, 2, 3, 6, 8, 13, 14, 20 (α = .85). **DP:** 5, 10, 11, 15, 22 (α = .58). **PA_SC:** 4, 7, 17, 21 (α = .49). **PA_EC:** 9, 18, 19 (α = .71). Correlations: r_EE*DP_ = .80; r_EE*PA_SC_ = .69; r_EE*PA_EC_ = .76, r_ PA_SC *PA_EC_ = .88; r_DP*PA_SC_ = .78 r_DP*PA_EC_ = .77	Results based on *n_2_*: **χ^2^** = 543.35; **df** = 164 **χ^2^/df** = 3.31; **RMSEA** = .08, **CFI** = .88	705 professionals (n_1_ = 350; n_2∶_355) from different occupational sector (healthcare, social and educational sector). Spain.
(8) 2006	Kanste *et al.*	EFA+CFA (ML)	**18 items** (deleted: 6, 13, 16, 22), **3 factors:** **EE:** 1, 2, 3, 8, 14, 20 (α = .87); **DP:** 5, 10, 11, 15 (α = .83); **PA:** 4, 7, 9, 12, 17, 18, 19, 21 (α = .81). Correlations: r_EE*DP_ = .35; r_EE*PA_ = −.31 r_DP*PA_ = not significant. N.B.:error term correlation of items 17 and 18.	**χ^2^** = 367; **df** = 131; **χ^2^/df** = 2.80; **RMSEA** = .05; **CFI** = .95	627 nurses and nurse managers. Finland.
(9) 2007	Vanheule *et al.*	CFA (ML) and partial measurement invariance	**20 items** (deleted: 12 and 16), **3 factors: EE:** 1, 2, 3, 6, 8, 13, 14, 20 (α = ni); **DP:** 5, 10, 11, 15, 22 (α = ni); **PA:** 4, 7, 9, 17, 18, 19, 21 (α = ni); Correlations: r_n1EE*DP_ = .6; r_n2EE*DP_ = .74; r_n1EE*PA_ = −.35; r_n2EE*PA_ = −.42; r_n1DP*PA_ = −.45; r_n2DP*PA_ = −.48	*n_1:_* **χ^2^** = 1171.68; **df** = 167; **χ^2^/df** = 7.02; **RMSEA** = .05; **CFI** = .93 *n_2_*_:_ **χ^2^** = 695.84 df = 167_;_ **χ^2^/df** = 4.17_;_ **RMSEA** = .04_;_ **CFI** = .92	Hospital nurses (n_1_ = 2515); nurses and assistants working in residential welfare institutions (n_2_ = 1639). Belgium.
(10) 2009	Poghosyan *et al.*	CFA (estimation method n.i.) + EFA	**22** **items, 3 factors:** **EE:** 1, 2, 3 8, 13, 14, 20 (α_USA_ = .93; α_Canada_ = .92;α_UK_ = .89; α_Germany_ = .86;α_New Zealand_ = .90; α_Japan_ = .88; α_Russia_ = .80 α_Armenia_ = .80); **PA:** 5, 6, 10, 11, 15, 16, 22 (α_USA_ = .78; α_Canada_ = .79; α_UK_ = .75; α_Germany_ = .79;α_New Zealand_ = .76; α_Japan_ = .82; α_Russia_ = .77 α_Armenia_ = .77); **DP:** 7, 9, 17, 18, 19, 21 (α_USA_ = .82; α_Canada_ = .82; α_UK_ = .80; α_Germany_ = .75;α_New Zealand_ = .82; α_Japan_ = .80; α_Russia_ = .71 α_Armenia_ = .36). Correlations: EE*DP: r_USA_ = .60 r_Canada_ = .59; r_UK_ = .57; r_Germany_ = .59; r_New Zeleand_ = .58; r_Japan_ = .60; r_Russia_ = .42; r_Armenia_ = .52; EE*PA: r_USA_ = −.25 r_Canada_ = −.24; r_UK_ = −.15; r_Germany_ = −.24; r_New Zeleand_ = −.17; r_Japan_ = .05; r_Russia_ = −.08; r_Armenia_ = .10; DP*PA: r_USA_ = −.33 r_Canada_ = −.32; r_UK_ = −.22; r_Germany_ = −.35; r_New Zeleand_ = −.27; r_Japan_ = −.10; r_Russia_ = −.24; r_Armenia_ = .12	U.S.: **χ^2^** = 19448; **df** = 206; **χ^2^/df** = 94.4;**RMSEA** = .09 Canada: **χ^2^** = 23292; **df** = 206; χ^2^/df = 113.06; **RMSEA** = .08 U.K.: **χ^2^** = 12217; **df** = 206; **χ^2^/df** = 59.30; **RMSEA** = .08 New Zealand: **χ^2^** = 5441; **df** = 206; **χ^2^/df** = 26.41; **RMSEA** = .08 Germany: **χ^2^** = 2420; **df** = 206; **χ^2^/df** = 11.74; **RMSEA** = .07 Japan: **χ^2^** = 7522; **df** = 206; **χ^2^/df** = 36.52; **RMSEA** = .08 Russia: **χ^2^** = 493; **df** = 206; **χ^2^/df** = 2.3; **RMSEA** = .06 Armenia:**χ^2^** = 723; **df** = 206; **χ^2^/df** = 3.5; **RMSEA** = .08	Nurse survey data from the US (13204), Canada (17403), UK (9855), Germany (2681), New Zealand (4799), Japan (5956), Russia (442), and Armenia (398)
(11) 2009	Kim & Ji	CFA (FIML) + longitudinal partial measurement invariance	**19 items** (deleted items: 2, 12, 16), **3 factors:** **EE:** 1, 3, 6, 8, 13, 14, 20 (α = n.i.). **DP:** 5, 10, 11, 15, 22 (α = n.i.). **PA:** 4, 7, 9, 17, 18, 19, 21 (α = n.i.). Correlations: r_EE*DP_ = n.i; r_EE*PA_ = n.i.; r_DP*PA_ = n.i.	**χ^2^** = 467; **df** = 149; **χ^2^/df** = 3.1; **RMSEA** = .07; **CFI** = .92	475 social workers interviewed in two occasions. California, US
(12) 2009	Oh & Lee	EFA (n_1_ = 124) + CFA(ML) (n_2_ = 125) validation on two subsamples obtained splitting the original sample by a random procedure	**15 items** (deleted: 6, 13, 14, 15, 16, 20, 21), **3 factors: EE:** 1, 2, 3, 8 (α = .90). **DP:** 5, 10, 11, 22 (α = .68); **PA:** 4, 7, 9, 12, 17, 18, 19 (α = .81) Correlations: r_EE*DP_ = n.i. r_EE*PA_ = n.i. r_DP*PA_ = n.i	**χ^2^** = 153.12; **df** = 87; **χ^2^/df** = 1.76; **RMSEA** = .08; **CFI** = .91	249 protective child service workers. South Korea.
(13) 2011	Còrdoba *et al.*	EFA + CFA(ULS)	**20 items** (deleted: 15 and 21). **3 factors:** **EE:** 1,2, 3, 6, 8, 13, 14,16, 20 (α = .83). **DP:** 5, 10, 11, 22 (α = .57). **PA:** 4, 7, 9, 12, 17, 18, 19 (α = .52).Correlations: r_EE*DP_ = n.i.; r_EE*PA_ = n.i.; r_DP*PA_ = n.i.	**χ^2^** = 286.522; **df** = 167; **χ^2^/df** = 1.7; **RMSEA** = .05; **CFI** = n.i.	314 healthcare professionals. Colombia.
(14) 2011	Chao *et al.*	EFA + CFA(PC)	**22 items** (no items deleted), **4 factors: EE:** 1, 2, 3, 6, 8, 13, 14, 16, 20 (α = .91). **DP_Ind**: 5, 15, 22 (α = .49). **DP_Rej**: 10, 11 (α = .63). **PA:** 4, 7, 9, 12, 17, 18, 19, 21 (α = .76). Correlations: r_EE*DP_ = n.i. r_EE*PA_ = n.i.; r_DP*PA_ = n.i.	**χ^2^** = 586.31; **df** = 203; **χ^2^/df** = 2.9; **RMSEA** = .074; **CFI** = n.i.	435 staff delivering direct care to persons with intellectual disability. New York State, US.
(15) 2013	Lee *et al.*	EFA (n_1_ = 949) + CFA (ML) (n_2_ = 897)	**20 items** (deleted: 14 and 22). **3 factors: EE:** 1, 2, 3, 6, 8, 13, 16, 20 (α = .91); **DP:** 5, 10, 11, 15 (α = .65); **PA:** 4, 7, 9, 12, 17, 18, 19, 21 (α = .86). Correlations: r_EE*DP_ = .66; r_EE*PA_ = .29; r_DP*PA_ = .38	*n_2_*: **χ2** = 599.95; **df** = 167; **χ2/df** = 3.59; **RMSEA** = .05; **CFI** = n.i.	1846 nurses. Taiwan
(16) 2013	Pisanti *et al.*	CFA (ML)	**20 items** (deleted: 12 and 16). **3 factors: EE:** 1, 2, 3, 6, 8, 13, 14, 20 (α = .88), **DP:** 5, 10, 11, 15, 22 (α = .70).**PA:** 4, 7, 9, 17, 18, 19, 21 (α = .83). Correlations: r_EE*DP_ = .49 r_EE*PA_ = −.14 r_DP*PA_ = −.39	**χ^2^** = 1155.23 df = 167; **χ^2^/df** = 6.9; **RMSEA** = .06; **CFI** = .92	1613 nurses. Italy.
(17) 2014	Mészáros *et al.*	CFA (ML) bifactor model in which all indicators load directly on an overall general factor (global burnout)	**1 factor**, **22 items (**α = n.i.)	**Satorra χ^2^** = 510; **df** = 187; **χ^2^/df** = 2.7; **RMSEA** = .052; **CFI** = .92	653 healthcare professionals (420 physicians and 233 nurses and nursing assistants). Hungary

Note: CFA = confirmatory factor analysis, EFA = exploratory factor analysis, ML = maximum likelihood estimation, WLS = weighted list squares estimation, ULS = unweighted list squares estimation, PC = principal components, FIML = full information maximum likelihood, EE = emotional exhaustion, DP = depersonalization, PA = personal accomplishment, PA_SC = self-competence, PA_EC = existential component, EE_PS = psychological strain, EE_SS = somatic strain, PA_S = personal accomplishment, PA_O = personal accomplishment others, DP_Ind = indifference about the care recipient, DP_Rej = rejection of the care recipient,α is the Cronbach coefficient of reliability and X^2^, RMSEA, and CFI are the principal fit indices published in every article considered, ni = not indicated.

The original version of MBI-HSS developed by Maslach and colleagues [3] has only been used in five of the 17 studies considered, as they were conducted on English-speaking samples (USA [8], [9], [10], [11]; Australia [12]). Two others involved Spanish-speaking samples (Spain [13]; Colombia [14]), five were conducted in cultural and linguistic North-European contexts (Netherlands [15]; Norway [16]; Sweden [17]; Finland [18]; Belgium [19]), one in Eastern Europe (Hungary [20]), and one in Southern Europe (Italy [21]). Finally, two studies were conducted on samples from the Far East (Taiwan [22]; South Korea [23]), and one was carried out on a number of heterogeneous samples of different cultural and linguistic origins [24].

Although most studies have obtained a factorial analysis similar to Maslach’s, the faithful reproduction of the model is not associated with entirely satisfactory fit indices in any of them. Such results have led several scholars to highlight some problematic aspects of the three sub-scales and to suggest that the original analysis must be reconsidered [3], [1]. To overcome the psychometric limits of the MBI-HSS, researchers [8]–[24] have proposed different and not always matching solutions that can be synthesized into four main procedures: allowing correlated error terms, allowing items to load on more than one factor, eliminating items and increasing or decreasing the number of factors.

The elimination of items is the most commonly adopted practice. Maslach [25] herself suggested eliminating items 12 (PA, “I feel very energetic”) and 16 (EE, “Working with people directly puts too much stress on me”). In their early studies, Maslach and colleagues found that item 12, intended to load on PA, measures EE, while item 16, intended to load on EE, tends to overlap with DP. Further studies [10], [13], [16], [17], [19], [21] have confirmed the results obtained by Maslach *et al.* [25], thus highlighting how fit indices considerably improve when items 12 and 16 are deleted.

A second research corpus detected other problematic items. Poghosyan *et al.* [24], in a study involving eight samples from the United States (U.S.), Canada, the United Kingdom (U.K.), Germany, New Zealand, Japan, Russia, and Armenia, highlighted that items 16 (EE) and 6 (EE) are weakly correlated with EE, while they strongly correlate with DP. The authors therefore approved of maintaining the 22-item factorial structure if items 6 and 16 are moved to the DP factor. On the North European side, a Finnish study [18] reported empirical evidence of the validity of an 18-item version in which items 6, 16, 13, and 22 are excluded, while in Belgium [19] a 16-item version eliminating items 1, 2, 5, 12, 14 and 19 was tested. A unique study conducted in South America [14] regarded items 15 and 21 as questionable. Moreover, in Eastern Asia (South-Korea), Oh and Lee [23] found empirical evidence for the validity of a three-factor, 15-item, version (excluding items 6, 13, 14, 15, 16, 20 and 21), whereas Lee *et al.* (Taiwan) [22] proposed a 20-item version, eliminating items 14 and 22.

One of the most disputed issues concerns the role of PA in the syndrome. In several studies PA was weakly correlated with the other dimensions that, in contrast, showed quite high correlations between them. This led Green *et al*. [26] to consider PA as less crucial than EE and DP, identified as the “core dimensions” of burnout. More recently, Kalliath *et al.* [8] gathered empirical evidence for a bi-dimensional version composed of EE and DP, containing only seven items.

Faced with unsatisfactory results, other scholars have attempted to conceptually reformulate the construct and suggested four – or even five – factor structures. Among these, Densten [12] proposed a five-factor model, in which EE and PA were divided, generating the components of *psychological strain*, *somatic strain*, *self-component* (self-perceived professional competence) and *others-component* (performance perceptions of others). Gil-Monte [13] suggested a four-factor solution in which, along with the EE and DP, two others dimensions originated from PA were added: the *self-competence* and the *existential component* linked to the interaction with patients. Similarly, Chao *et al.* [11] explored a four-factor structure dividing PA into *indifference toward patients* and *rejection of the recipients*.

In sum, these contrasting results on MBI-HSS psychometric properties warrant several considerations. It is well known that the MBI was developed in the North-American context, and it is therefore probable that the absence of systematic results may be caused by the linguistic and cultural heterogeneity of the samples on which the model was tested. Items may indeed assume different meaning depending on the context in which they are presented (according to Maslach *et al.* [27] North Americans may be more likely than Europeans to give “extreme answers” to items or to express cynicism), or because occasionally something is “lost in translation” [28].

Psychometric studies with Italian participants are rare. Sirigatti and Stefanile [29], [30], [31], [32], who edited the adaptation of the inventory for Italy in the 1990s, suggested that different models had to be tested in order to determine the most suitable factorial solution, but this proposal has never been developed. Moreover, exploratory factorial analyses conducted on heterogeneous samples in relation to Italian occupational sectors have highlighted that the structure proposed by Maslach, however reachable by imposing the three factors, is not always the most adequate.

The aim of the present study is then to examine the factor structure and the psychometric properties of the MBI-HSS and to gain insight into the functioning of the MBI-HSS in Italy as follows:

- evaluating the functioning, i.e., reliability and validity, of the MBI-HSS items with regard to an Italian sample;
- testing the main alternative MBI-HSS models in order to identify the most appropriate model to measure burnout.

The study includes the comparison of ten different models: the original Maslach’s model specification [1], the first theoretical, relevant revision of the model proposed by Green *et al.* in the 1990s [26] and eight of those previously reviewed (identified as numbers 2, 3, 7, 8, 11, 13, 14, and 15 in Table 1). These eight models have been considered for the present study because they A) avoid covariances between error terms, B) avoid cross loading items, and C) imply the elimination of a maximum of four items. Including covariances between error terms implies admitting problems in item phrasing, which can result in response bias – such as acquiescence or impression management [33], [34], [35] – or lexical redundancy in items wording and specification, or item redundancy [36], [37]. Specifying models with cross loading items on multiple factors compromises their integrity [38]. Moreover, in trying to measure a multidimensional construct, each factor’s content coverage in the measure must be preserved. Each deleted item causes a loss of content validity, and the more items that are deleted, the more the content coverage is compromised. An abbreviated scale can result in a different, alternative assessment that does not measure what it originally intended to measure [39].

Table 2 presents the ten selected models for the comparison. Each model is identified by an alphanumeric label composed of the number of factors included in the model (2–5) and a letter (A–E) identifying the number of items within each factor when the number of latent dimensions remains stable but the set of considered items does not.

**Table 2:** Original and alternative measurement of MBI-HSS: items and model specifications.

*Model*	M2	M3	M3A	M3B	M3C	M3D	M3E	M4	M4A	M5
*Author(s)*	Green*et al*	Maslach*et al.*	Schaufeli*et al.*	Kimand Ji	Kanste*et al*.	Còrdoba*et al*.	Lee*et al.*	Chao*et al*	Gil-Monte	Densten
*Year*	1991	1986	2001	2009	2006	2011	2013	2011	2005	2001
*Included* *items*	*22*	*22*	*20*	*19*	*18*	*20*	*20*	*22*	*20*	*19*
*Dimensions* *Items*	*2*	*3*	*3*	*3*	*3*	*3*	*3*	*4*	*4*	*5*
1 (EE)	CoreB	EE	EE	EE	EE	EE	EE	EE	EE	EE_SS
2 (EE)	CoreB	EE	EE	/	EE	EE	EE	EE	EE	EE_SS
3 (EE)	CoreB	EE	EE	EE	EE	EE	EE	EE	EE	EE_SS
4 (PA)	PA	PA	PA	PA	PA	PA	PA	PA	PA_SC	PA_S
5 (DP)	CoreB	DP	DP	DP	DP	DP	DP	DP_Ind	DP	DP
6 (EE)	CoreB	EE	EE	EE	/	EE	EE	EE	EE	EE_PS
7 (PA)	PA	PA	PA	PA	PA	PA	PA	PA	PA_SC	PA_O
8 (EE)	CoreB	EE	EE	EE	EE	EE	EE	EE	EE	EE_SS
9 (PA)	PA	PA	PA	PA	PA	PA	PA	PA	PA_EC	PA_S
10 (DP)	CoreB	DP	DP	DP	DP	DP	DP	DP_Rej	DP	DP
11 (DP)	CoreB	DP	DP	DP	DP	DP	DP	DP_Rej	DP	DP
12 (PA)	PA	PA	/	/	PA	PA	PA	PA	/	/
13 (EE)	CoreB	EE	EE	EE	/	EE	EE	EE	EE	/
14 (EE)	CoreB	EE	EE	EE	EE	EE	/	EE	EE	/
15 (DP)	CoreB	DP	DP	DP	DP	/	DP	DP_Ind	DP	DP
16 (EE)	CoreB	EE	/	/	/	EE	EE	EE	/	EE_PS
17 (PA)	PA	PA	PA	PA	PA	PA	PA	PA	PA_SC	PA_O
18 (PA)	PA	PA	PA	PA	PA	PA	PA	PA	PA_EC	PA_S
19 (PA)	PA	PA	PA	PA	PA	PA	PA	PA	PA_EC	PA_S
20 (EE)	CoreB	EE	EE	EE	EE	EE	EE	EE	EE	EE_PS
21 (PA)	PA	PA	PA	PA	PA	/	PA	PA	PA_SC	PA_O
22 (DP)	CoreB	DP	DP	DP	/	DP	/	DP_Ind	DP	DP

Note: In labels M2 and M3 to M5, the counter emphasize the number of factors, while the suffix A, B, or C identifies the number of items.

A slash represents the items excluded from the specified models.

CoreB = core dimension of burnout, EE = emotional exhaustion, DP = depersonalization, PA = personal accomplishment, DP_Ind = indifference about the care recipient, DP_Rej = rejection of the care recipient, PA_SC = self-competence, PA_EC = existential component, EE_PS = psychological strain, EE_SS = somatic strain, PA_S = personal accomplishment, PA_O = personal accomplishment others.

## Materials and Methods

### Data collection: participants, procedures, and instrument

Data were collected during a multi-center intervention study conducted in five hospitals in Northwestern Italy between 2010 and 2012. The research conformed to the provisions of the Declaration of Helsinki in 1995 (as revised in Edinburgh 2000), and all ethical guidelines were followed as required for conducting human research, including adherence to the legal requirements of Italy. The research project was approved by the *Hospital Board of Directors* of the five hospitals involved in the study: *Cardinal Massaia* (Asti); *SS. Annunziata* (Savigliano, Cuneo); *San Giovanni Bosco, Gradenigo,* and *Maria Vittoria* (Turin). Additional ethical approval was not required since there was no treatment including medical, invasive diagnostics or procedures causing participants psychological or social discomfort, nor were patients the subject of data collection. With the Hospital Board of Directors’ approval, department chiefs and nurse coordinators from each ward were asked for authorization to administer the questionnaire to the nurses. Participants volunteered in the research without receiving any reward and were not asked to sign consent forms, but the questionnaire return implied consent. The cover sheet clearly explained the research aim, the voluntary nature of participation, the anonymity of the data, and the elaboration of the findings.

Participants represented a sufficiently large and heterogeneous sample of the nursing staff employed in Northwestern Italy. The sample consisted of 925 operators, mainly women (66.6%), with a mean age of 37.9 years (SD 8.8), employed in the health sector for an average time of 14.4 years (SD 9.7) and actually working in emergency (37.8%), medical (25%), surgical (13.2%), mental health (9%), diagnostic (6.8%), maternity and infant (30%), or in ambulatory wards (4.3%).

Data were collected through a self-administered questionnaire including:

background indicators of socio-demographic and professional attributes of participants;
the Italian version of the MBI-HSS [30], 22-item assessment with a seven-point frequency rating scale ranging from 0 (“never”) to 6 (“every day”).

### Statistical analysis

The psychometric properties of the Italian version of MBI-HSS with reference to a sample of nurses were preliminarily examined performing an item analysis.

Total and sub-scale reliabilities were assessed by means of Cronbach’s coefficients, while the contribution to internal consistency at the level of single items was evaluated through item-total and item-subscale correlations.

In order to check the discriminatory capacity of each item, a procedure analogous to the one carried out by Lee *et al.* [22] was performed. The authors suggest calculating the critical ratio, i.e., the t-value obtained when comparing the means between two groups, the lower 27^th^ and the upper 73^rd^ percentile of the score distributions in the sample. In the present study, the group definition based on percentiles is maintained, but each item discrimination in relation to its theoretical sub-dimension is studied using anova decomposition to represent it in terms of effect size.

Item evaluation also included considerations about response distributions, i.e., their normality and multi-normality. In the sample, items do not show a multivariate normal distribution. Therefore, the Prelis package was used to compute an asymptotic covariance matrix to correct the ML estimations obtained by the Lisrel software, 8.72 version [40]. Since in previous studies no agreement was reached on factor relations, each model was estimated with both orthogonal and oblique factorial specifications.

The model evaluation and comparison were conducted using both incremental and absolute fit indices: the comparative fit index (CFI) [41], nonnormed fit index (NNFI) [42], or alternatively, the Tucker-Lewis index (TLI) [43], root mean square error of approximation (RMSEA) [44] and standardized root mean squared residual (SRMR) [45], [46]. Since these indices are widely used, their presentation facilitates comparison with previous results. Moreover, they are the most sensitive in distinguishing good models from poor ones, with misspecified factor loadings or/and factor covariances. Following Hu and Bentler [47], a cut-off value ≥.95 for CFI, a cut-off value ≤0.6 for RMSEA, and ≤0.8 for SRMR are an efficient strategy to evaluate model fit. Furthermore, the consistent Akaike information criterion (CAIC) [48] and expected cross-validation index (ECVI) [49], to compare non-nested models, and the Satorra and Bentler scaled difference (SB-Diff) to test the differences between nested models [50], [51] were used.

## Results

### Reliability and item analysis

The reliability of all items measured by Cronbach’s α index is 0.800. The only item with strong inhomogeneity with reference to the whole scale is item 12 (PA, Cronbach’s α, if the item is deleted = 0.822) with a negative item-total correlation (−0.130). Cronbach’s α for the sub-scales is 0.896 for EE, 0.755 for DP, and 0.821 for PA. EE items show quite high item-total correlation; the lowest correlation of the set is item 16 (0.524), whose deletion would not modify the internal homogeneity of the subset (Cronbach’s α, if the item is deleted = 0.894). PA and, above all, DP item-total correlations are weaker, but no deletion would leave the two sub-scales unaltered nor increase their homogeneity.

All anova tests performed on sub-dimensions are significant, which means that all items in each set can discriminate between the relative score extremes. Nevertheless, the value of η^2^ coefficient associated with each item signals a different item performance. For every sub-dimension, η^2^ is the proportion of score variability accounting for between-group differences after dividing participants into more and less exhausted, depersonalized, or accomplished groups (Table 3). We can interpret η^2^ as items discriminatory power and even if significant, items 16 (EE) and 15 (DP) are least capable of discriminating between participants.

**Table 3:** MBI_HSS item homogeneity, discrimination, and distributional form.

	Corrected item-total correlation	α if item deleted	Sub-scales corrected item-total correlation	Sub-scales α if item deleted	η^2^	S	K	Kolmogorov Smirnov
EE1_1	.573	.784	.729	.879	.727	0.248	−0.975	.161
EE2_2	.539	.786	.647	.886	.654	−0.235	−0.974	.162
EE3_3	.496	.788	.632	.887	.652	0.195	−1.028	.145
EE6_4	.539	.787	.604	.889	.561	0.824	−0.219	.225
EE8_5	.592	.782	.765	.876	.800	0.552	−0.911	.222
EE13_6	.575	.784	.704	.881	.674	0.864	−0.250	.227
EE14_7	.568	.783	.615	.889	.676	0.183	−1.080	.141
EE16_8	.472	.790	.524	.894	.442	1.050	0.360	.242
EE20_9	.544	.785	.708	.881	.648	1.133	0.126	.265
DP5_1	.318	.798	.537	.707	.579	1.301	0.766	.286
DP10_2	.388	.794	.623	.671	.746	1.139	0.143	.255
DP11_3	.385	.794	.563	.696	.703	0.974	−0.215	.244
DP15_4	.308	.798	.475	.728	.420	1.841	2.731	.344
DP22_5	.348	.796	.424	.745	.502	1.289	0.669	.259
PA4_1	.246	.802	.465	.807	.527	−0.737	−0.235	.199
PA7_2	.252	.801	.602	.788	.644	−1.164	0.836	.219
PA9_3	.189	.805	.492	.803	.568	−0.822	−0.160	.219
PA12_4	−.130	.822	.460	.809	.549	−0.439	−0.809	.176
PA17_5	.249	.801	.662	.781	.677	−0.957	0.368	.240
PA18_6	.060	.810	.616	.786	.687	−0.639	−0.413	.214
PA19_7	.142	.807	.495	.803	.535	−0.587	−0.439	.180
PA21_8	.141	.807	.523	.799	.632	−0.749	−0.321	.214

Note: EE = emotional exhaustion, DP = depersonalization, PA = personal accomplishment; S = Skewness, K = Kurtosis.

All η^2^ values are significant, p<0.000. All Kolmogorov-Smirnov values are significant, p<0.000; Lilliefors correction.

Items do not show normal distributions. Tests of multivariate normality in the Prelis application confirm the non-normality of items distribution: the reported normality chi-square test strongly rejects the null hypothesis (χ^2^ = 3639.7, p<0.000).

### Model comparison using CFA

Two baseline models were used to obtain an exhaustive view of model performance analysis: M0, representing a null model, with no covariances between items, and M1 a unique dimension model of burnout. Model fits are presented (Table 4) in order of factor cardinality, starting from M0 to M5 (five latent dimensions), and in order of item cardinality within each specification. Furthermore, each model occupies two rows because of the rotation specification: orthogonal or oblique.

**Table 4:** Alternative models of MBI_HSS: fit indices.

	Item	Rot.	mff X^2^	Norm. X^2^	df	SB X^2^	RMSEA	RMSEA CI	SRMR	CFI	NNFI	ECVI	CAIC
M0	22		8250.13	18467.95	231	15830.21	0.27	0.27; 0.27	0.28	0.00	0.00	20.03	18640.20
M1	22		3523.39	5857.23	209	4813.07	0.15	0.15; 0.16	0.14	0.70	0.67	6.43	6201.75
M2(Green 1991)	22	ort	1879.50	2438.85	209	1951.04	0.09	0.09; 0.10	0.10	0.89	0.88	2.73	2783.36
		obl	1860.74	2436.77	208	1944.16	0.09	0.09; 0.10	0.09	0.89	0.88	2.73	2789.11
M3(Maslach 1986)	22	ort	1682.13	1751.11	209	1414.84	0.08	0,08; 0,08	0.15	0.92	0.91	2.00	2095.63
		obl	1347.22	1473.57	206	1174.96	0.07	0.07;0–08	0.08	0.94	0.93	1.70	1841.57
M3A(Schaufeli 2001)	20(12,16)	ort	1217.61	1230.75	170	1008.23	0.07	0.07; 0.08	0.14	0.93	0.93	1.42	1543.94
		obl	910.60	962.12	167	777.62	0.06	0.06; 0.07	0.06	0.95	0.95	1.13	1298.81
M3B(Kimand Ji 2009)	19(2, 12, 16)	ort	997.15	974.84	152	792.23	0.07	0.06; 0.07	0.14	0.94	0.93	1.14	1272.37
		obl	675.81	699.60	149	559.85	0.06	0.05; 0.06	0.06	0.96	0.96	0.85	1020.62
M3C(Kansteet al. 2006)	18(6, 13, 16, 22)	ort	1044.88	1083.17	135	891.77	0.08	0.07; 0.08	0.13	0.92	0.91	1.25	1365.05
		obl	809.72	840.36	132	683.76	0.07	0.06; 0.07	0.07	0.94	0.93	0.99	1145.72
M3D(Cordobaet al. 2011)	20(15, 21)	ort	1502.68	1568.33	170	1264.98	0.08	0.08; 0.09	0.15	0.92	0.91	1.78	1881.52
		obl	1191.69	1293.15	167	1027.51	0.08	0.07; 0.08	0.08	0.94	0.93	1.49	1629.83
M3E(Lee etal. 2013)	20(14, 22)	ort	1524.15	1609.99	170	1299.83	0.09	0.08; 0.09	0.15	0.91	0.90	1.83	1923.18
		obl	1212.33	1340.29	167	1066.23	0.08	0.07; 0.08	0.08	0.93	0.92	1.54	1676.97
M4(Chaoet al. 2011)	22	ort	1883.87	2104.73	208	1714.55	0.09	0.09; 0.09	0.16	0.90	0.89	2.38	2457.07
		obl	1296.74	1419.87	203	1132.15	0.07	0.07; 0.07	0.08	0.94	0.93	1.65	1811.09
M4A(Gil-Monte2005)	20(12, 16)	ort	1606.89	1532.68	170		0.08	0.08; 0.09	0.16	0.91	0.90	1.75	1845.87
		obl	843.05	883.76	164	713.12	0.06	0.06; 0.07	0.06	0.96	0.95	1.06	1243.93
M5(Densten2001)	19(12, 13, 14)	ort	2349.74	2096.88	152	1801.16	0.10	0.10; 0.10	0.21	0.85	0.83	2.35	2394.41
		obl	745.44	753.41	142	608.91	0.06	0.06; 0.06	0.05	0.96	0.95	0.92	1129.24

Note: In the labels, M1, M1 to M5, the counter emphasizes the number of factors, while the suffix A, B, or C identifies the number of items.

The results show that, whatever the dimensionality specified, oblique solutions are better than orthogonal ones: factor covariance yielded an appreciable increase in fit, and the Satorra-Bentler scaled difference is always significant (Table 5), suggesting that the relationship between the construct sub-dimensions cannot be ignored.

**Table 5:** Satorra-Bentler scaled chi-squared statistics for nested CFA models.

	M2obl			M3obl			M4obl			M3Aobl			M4Aobl		
	SB diff	df	p	SB diff	df	p	SB diff	df	p	SB diff	df	p	SB diff	df	p
M2ort	4.56	1	0.03												
M3ort				3998.21	3	0.00									
M4ort							1972.67	6	0.00						
M3Aort										725.08	3	0.00			
M4Aort													3647.43	6	0.00

Based on the oblique specification results shown in Table 4, Maslach’s model (M3) shows somewhat satisfactory performance, as fit indices do not reach the anticipated cutoffs: CFI is 0.94, RMSEA is 0.07, and SRMR is 0.08.

Complicating the model by adding a latent dimension to structure the original 22-item set (M4) does not yield substantial benefits. The Satorra-Bentler scaled difference is significant (SB-Dif between M3 and M4 = 42.93, df = 4, p<0.000), but fit indices remain stable.

**Figure 1:**
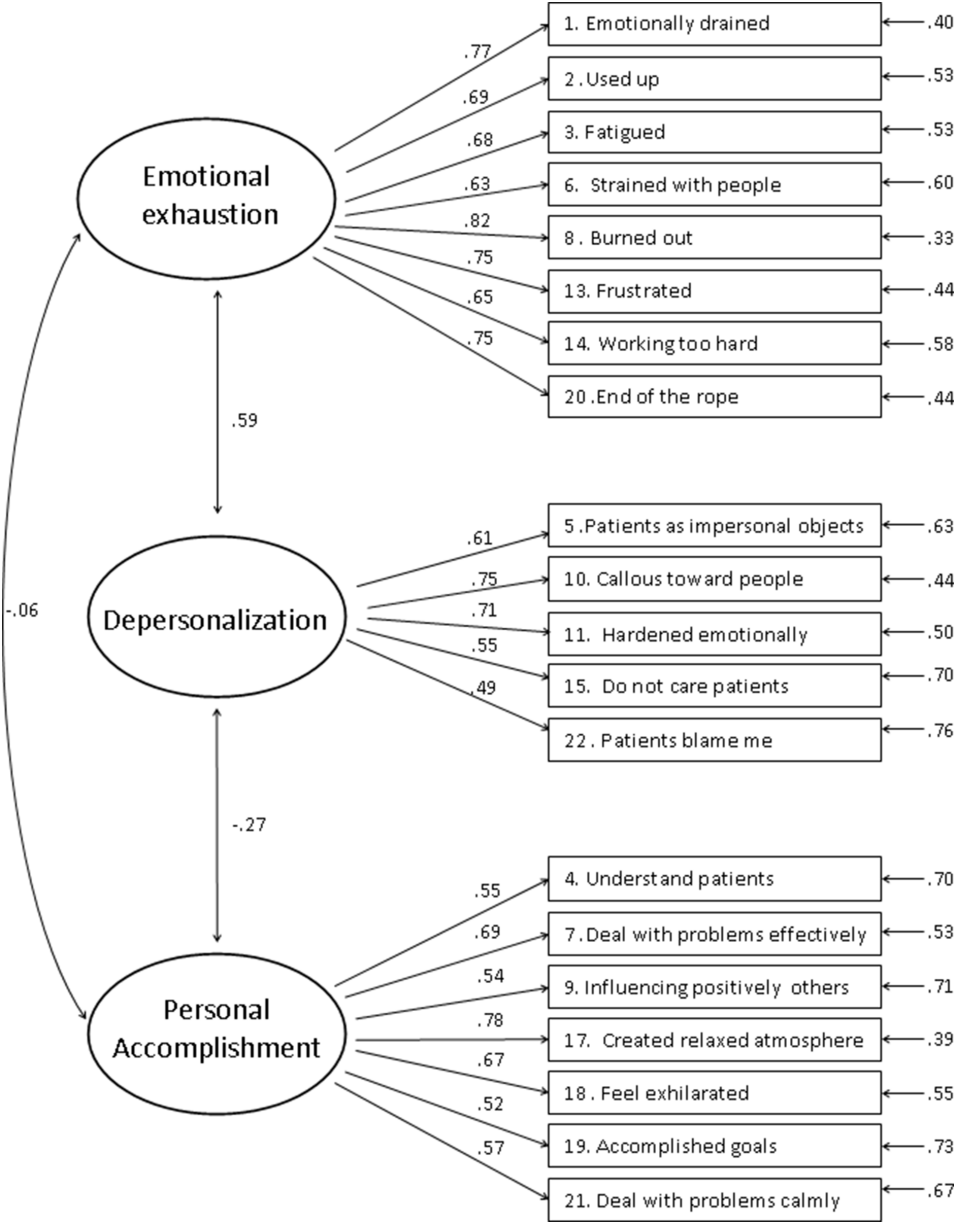
Confirmatory Factor Analysis of Italian Version of the MBI-HSS (20 items, excluding 12 and 16).

The three-dimensional models (M3A, M3C, and M3D), which preserve the original structure by deleting two items, do not show better features. The only solution that shows an increase compared to Maslach’s is the one proposed by Schaufeli *et al.* [15], yielding a CFI of 0.95, and 0.06 for both RMSEA and SRMR. The model is more parsimonious (CAIC = 1298.91) and shows better expected cross validation (ECVI = 1.13). In this model (M3A), illustrated in Fig. 1, all parameters are associated with satisfying estimates and correlations between sub-scales represent a well-known profile in Italy [52], [53], [54]: EE and DP are positively associated and both have a weak negative correlation with PA.

The loss of information caused by item elimination yields a measurement benefit only if the deleted items are 12 and 16, as pointed out by Schaufeli *et al.* [15], and often observed in various national contexts, including Italy [21]. The results of this model (M3A) confirm the indications of the item analysis conducted on the data reported in this study, showing the criticality of item 12, having a general and ambiguous formulation, and of item 16, the worst indicator in the item set relative to EE. Cordoba *et al.* (M3D) [14] proposed to delete items 15 and 21, while Lee *et al.* (M3E) [22], omitted items 14 and 22 but in the present sample these specifications are detrimental compared to the Schaufeli’s model (M3A). For both models, the discrepancies between observed and estimated response covariances measured by SRMR and RMSEA increase to 0.8 and, conversely, CFI decreases to 0.94 and 0.93. The same applies to the model suggested by Kanste *et al.* (M3C) [18]. The deletion of four items inevitably yields better parsimony (CAIC = 1145.72) but does not significantly increase its fit. M3C performance is similar to Maslach’s but worse than Schaufeli’s model.

Gil-Monte’s specification (M4A) [13], defined by splitting the same set of items selected by Schaufeli *et al.* [15] into a four-factor structure, where PA is divided into self-competence and existential components, increases fit in terms of chi-square (SB-Dif = 73.96, df = 3, p<0.000) and CFI (0.96), but not RMSEA and SRMR. There is no complete evidence that M4A outperforms M3A and, as Gil-Monte himself concludes, it is convenient to maintain the three-dimensional model because the correlation between the two factors is consistent (r>0.80), and these are clearly aspects of the same dimension [13].

Densten (M5) [12], continuing to increase complexity, separates psychological strain from somatic strain, distinguishes between personal accomplishment related to self and others, and eliminates three items (12, 13, and 14). This five-factor oblique model seems to be a good interpretation of the between-item covariance in terms of model fit (particularly considering CFI = 0.96 and SRMR = .05), but the results include a quasi-perfect correlation between the two PA factors (self and others) and a correlation of .87 between the two EE dimensions (psychological and somatic strain). Densten’s model includes a theoretically interesting suggestion for EE, although obtained by forcing items. To reach the distinction between psychological and physical stress, one is compelled to eliminate two items (13 and 14) that, on the contrary, prove to be completely homogeneous and discriminating for EE. Moreover, the remaining items are re-aggregated, forcing their meaning: although item 1 states “I feel *emotionally* drained from my work” it is attributed to *somatic* strain.

M3B has the best model fit (CFI is 0.96, RMSEA 0.06, and SRMR 0.06). Kim and Ji [10] further reduced the items set by eliminating items 12 and 16, as in Schaufeli’s model (M3), and item 2 due to its high covariance of error terms with item 1. Such covariance was not high in the present research dataset. Rather, item 2 yields the highest number of residuals between the covariances observed and those reproduced by the model: deleting this item from the set means eliminating the observed covariances that could not be explained on the basis of the three esteemed latent constructs. Examining item-error covariances in the Italian nurses sample, the highest one refers to the 10–11 couple (both DP items), and there are non-negligible item-error covariances between items 5 and 6 (respectively, DP and EE items), 17 and 18, and 18 and 19 (both couples on PA). In all cases, in the Italian version the item pairs contain lexical redundancy, are contiguous and refer to the same construct, with the exception of items 5 and 6, in which the formulation of item 6 is entirely compatible with a double loading on EE and DP. Therefore, it seems that the order of item presentation as well as item lexical noise and redundancy [36], [37] may contribute to the level of observed covariances. However these covariances cannot be explained by the substantive dimensions of the burnout construct.

## Discussion and Conclusions

Analyses performed on the Italian sample showed that A) the factorial structure underlying the MBI-HSS is three-dimensional and follows Maslach’s model, even if B) the item set is the one suggested by Kim and Ji [10], deleting items 2, 12 and 16, or, preferably, by Schaufeli *et al.* [15] deleting only items 12 and 16.

Results confirm the original construct dimensionality and subdimensions meaning, even if the three dimensional specification is compared to more complex (in terms of numbers of latent dimensions) or simpler (in terms of numbers of considered items) specifications, that might be more satisfying because increasing construct dimensionality or deleting items facilitate data reproduction and generally improve model fit. Alternative models of MBI-HSS considered in this study do not yield results that justify a redefinition of valid items, an excessive shortening of the scale or, even more, a theoretical redefinition of the syndrome.

In sum, a conclusive view of the inventory is that it actually measures three dimensions and that every data-driven re-specification of the model could result in an attempt to solve, *a posteriori* and by means of structural equations, problems not regarding construct validity but item redundancy, their lexical noise, desirability, or not less crucially, the order of item administration.

Potential limitations of the current study are the non-probabilistic nature of the sample, that is not intended to be representative, and the fact that this study was not specifically designed with the purpose of studying response style or bias potentially associated with MBI-HSS items, as items performances presume to be. Present data did not permit the control and estimation of the possible effect of items characteristics and order of presentation on participants’ answers.

Considering the relevance and the worldwide diffusion of the MBI, further studies should: A) focus on the order of items presentation so as to determine if and how this aspect affects the functioning of the items and the scale; B) identify and rephrase those items which show bad-functioning across different linguistic and cultural contexts; C) rephrase items of the Italian version that sound excessively similar or that show meaning redundancy (i.e., the coupled items 6/16 and 10/11).
